# Different sedentary behavior domains present distinct associations with eating-related indicators

**DOI:** 10.1186/s12889-024-17760-2

**Published:** 2024-01-23

**Authors:** Pedro B. Júdice, Eliana V. Carraça, Inês Santos, António L. Palmeira, Flávio Jerónimo, Marlene N. Silva

**Affiliations:** 1 CIDEFES, Universidade Lusófona, Lisboa, Portugal; 2 Laboratório de Nutrição, Faculdade de Medicina, Centro Académico de Medicina de Lisboa, Universidade de Lisboa, Lisboa, Portugal; 3 Instituto de Saúde Ambiental (ISAMB), Faculdade de Medicina, Universidade de Lisboa, Lisboa, Portugal; 4 Programa Nacional para a Promoção da Atividade Física - Direcção-Geral da Saúde, Lisboa, Portugal

**Keywords:** Eating behavior, Eating patterns, Mediterranean diet, Physical activity, Sedentary behavior

## Abstract

**Background:**

Unhealthful dietary patterns have been consistently associated with low levels of physical activity (PA), but studies dedicated to sedentary behavior (SB) are scarce, especially in adults. The few studies that investigated the association between SB and dietary patterns focused mostly on specific types of SB, such as TV-watching or screen time. SB can be accumulated in distinct domains (i.e., work, transport, and leisure-time), thus, it is key to investigate in depth the impact that different domains of SB can have on eating-related indicators. We aimed to investigate the associations between different SB domains and eating-related indicators, in a sample of adults.

**Methods:**

Cross-sectional data from students, teachers, and staff from a Portuguese University was collected in November/2021 through an anonymous online survey. Data analyses were performed using the IBM SPSS software (version 28.0) and included descriptive statistics, partial correlations, and group comparisons using one-way ANOVA. Daily average SB at work/study, transport, and in leisure-time were self-reported and eating-related indicators were measured with several items from the Mediterranean Diet Score. Specific eating-related behaviors reflecting a protective eating pattern (e.g., eating breakfast regularly), and eating behavior traits (e.g., external eating) were also assessed. Body mass index (BMI) was calculated as weight (kg)/height(m)^2^. The International Physical Activity Questionnaire/Short-Form was used to assess PA.

**Results:**

The sample included 301 adults (60.1% women), with a mean age of 34.5 years. Overall, leisure-time SB was inversely associated with adherence to the Mediterranean diet (*r* = -0.20; *p* < 0.001) and with a protective eating profile (*r* = -0.31; *p* < 0.001). Higher transport SB was also related to lower adherence to the Mediterranean diet (*r* = -0.20; *p* < 0.001) and to an unhealthier eating profile (*r* = -0.22; *p* < 0.001), but no associations were found for work-related SB (*p* > 0.05). These results persisted after the adjustment for BMI, sex, and self-reported PA. These results were impacted by the age tertile.

**Conclusions:**

Our findings suggest that adults with higher levels of SB in leisure-time and transport domains tend to report less healthy eating-related behaviors, irrespective of BMI, sex, and PA level. However, some differences in these associations were found according to the age tertile. This information may assist public health authorities in focusing their efforts in augmenting literacy on SB, namely on how SB can be accumulated via different settings. Furthermore, public health literacy efforts need to extend besides the more known deleterious effects of SB on health (e.g., diabetes, cardiovascular disease), to also include the interplay with eating indicators. Strategies to reduce SB and unhealthy eating should be particularly focused on promoting physically active forms of commuting and reducing SB in the leisure setting.

## Introduction

Unhealthy diet and poor eating behaviors can contribute to lifestyle linked conditions such as obesity [1], cardiovascular diseases, type 2 diabetes [2], and some types of cancer [3]. The mechanisms underlying the association between a higher physical activity (PA) level and a healthier diet are far from being settled [3], but it seems that PA is generally positively associated with a healthier diet and with improved eating choices [4–6], a reduced reinforcing value of high energy dense foods compared to non-active controls [7], and with improved eating behavior traits, like higher eating self-efficacy, and lower emotional and external eating [8, 9].

However, some contradictory findings regarding the association between PA and healthy eating have also been reported [10]. This might be related with the interference of sedentary behavior (SB), which usually occupies the majority of our 24-h day cycle. SB refers to any waking behavior characterized by an energy expenditure of less than 1.5 METs, in a seated/reclined position [11] and current evidence supports the notion that SB displaces light intensity PA (LIPA) [12]. Excessive time in SB is independently associated with several non-communicable diseases [13, 14], and even with higher mortality [13, 15], thus, there is a possibility that SB may play a specific role in people’s diet and eating behaviors, that can somehow alter the association between PA and these important outcomes.

Regarding diet, a systematic review found that SB in children, adolescents, and adults was associated with elements of a less healthier diet, including lower fruit and vegetable consumption, higher consumption of energy-dense snacks, drinks, fast food, and a higher total energy intake [16]. This seems especially observed in younger populations [17]. Also, regardless of the use of objective or subjective measurements, older adults without a healthy diet appear to engage in a more inactive and sedentary lifestyle [18]. Screen time has also shown negative correlations with the intake of breakfast, vegetables, and fruit [19]. Furthermore, a review suggested that SB was consistently identified as an important correlate of dietary behavior [20] and recent evidence suggests that displacing SB with LIPA improves dietary quality in older females, and SB fragmentation seems valuable for various dietary outcomes [21]. Although limited, there is some evidence suggesting that SB might also be impactful on eating behavior traits. One recent investigation found that decreased cognitive impulse control following after-school sedentary screen time may be associated with increased brain activation for high energy dense foods, possibly contributing to disinhibited eating [22].

SB incorporates several domains (e.g., work, leisure-time, and transport related SB), and to the authors best knowledge evidence on the associations between distinct SB domains and eating behaviors is scarce [23, 24]. One investigation including participants from five urban regions in Europe found that domain-specific SBs were related to unhealthy dietary behaviors, except for transport related SB [23]. Furthermore, the fact that almost none of the associations were moderated by age or gender suggests that these associations may hold across age and gender groups [23]. A more recent investigation [24] found no relationship for high SB with dietary and lifestyle habits in adjusted analyses, however, breaks in SB at work were associated with high consumption of dairy products and cereals. Also, more breaks from SB at leisure-time were associated with higher consumption of fruits and vegetables, even among the ones with higher SB [24].

Though the available evidence supports the putative role of PA and, to a smaller extent, the detrimental role of SB on eating-related behaviors, prior research has focused mostly on isolated macronutrient categories within a diet, neglecting the potentially interactive effects of multiple diet components and related behaviors. No studies addressing the associations between SB domains and eating behavior traits were found. Indeed, behind one’s food choices and consumption, there are several behavior traits that explain the higher or lower capacity someone has, to self-regulate eating and one’s food choices in the current obesogenic food environment [25]. Thus, it is important that PA and/or SB effects on eating indicators are addressed in a more comprehensive way.

In the present investigation and considering the limited evidence available, we aimed to investigate the cross-sectional associations between distinct SB domains (i.e., work, leisure-time, and transport) and several eating-related indicators (i.e., dietary intake, eating habits’ protective profile, and eating behavior traits) in adults. Additionally, we aim to explore if these relationships are independent of sex, age, body mass index (BMI), and PA levels.

## Materials and methods

### Study design & participants

The present study has a cross-sectional design and is based on self-reported data from 301 participants (students, teachers, and staff) from a Portuguese University. This project was approved by the Ethics Committee of Lusófona University (Reference: ILIND/F + /EI/02/2020), and all participants gave their consent prior to participation. Participants were recruited via institutional emails, the website of the project, and social networks and invited to fulfill an anonymous online survey using a *Qualtrics™ link* during November 2021. The only inclusion criterion was related to being a staff member, teacher, or student at the university. The survey took approximately 20 min to complete. Participants could complete the questionnaire until the end of November.

### Measures

#### Sociodemographic

The participants were asked about their age (years), height (m) and weight (kg). Body mass index (BMI) was calculated using the ratio weight (kg)/height (m)^2^.

#### Sedentary behaviors

For assessing SB domains, the participants were asked to indicate, “The time spent on a regular weekday and weekend day while: 1) seated working/studying without screen; 2) seated working/studying with screen; 3) seated watching TV/computer/tablet/phone for leisure; 4) seated doing a hobby like playing an instrument, playing cards, reading; 5) seated in transport (private or public)”. A daily weighted average of SB at work/study was calculated based on the sum of 1) and 2), considering the number of weekdays and weekend days. Daily transport-related SB was calculated as the weighted average of the number of weekdays and weekend days. A daily leisure-time SB was calculated by summing 3) and 4) and obtaining the weighted average of the number of weekdays and weekend days [26, 27].

#### Physical activity

The International Physical Activity Questionnaire Short-Form (IPAQ-SF) was used to assess PA [28]. The IPAQ questionnaire is formed of seven questions related to specific types of PA e.g., walking, moderate, and vigorous activities, in terms of weekly frequency and duration of each specific type of activity. The Total PA (min/week) was calculated by summing the marked minutes per week spent on walking, moderate, and vigorous activities [28].

#### Mediterranean diet adherence

The MedDietScore measures adherence to the Mediterranean Diet [29]. Briefly, the MedDietScore is a composite 11-item index that examines the weekly intake of 9 food groups, namely non-refined cereals, fruits, vegetables (raw or cooked), legumes, potatoes, fish (and fisheries), meat and meat products, poultry, full fat dairy (including cheese), as well as olive oil use, and alcohol drinking, based on the principles of the traditional Mediterranean Diet. Each response given for an item of the index is assigned a score from 0 to 5 (never, rare, frequent, very frequent, weekly, and daily consumption) based on the rationale of the traditional dietary pattern (or on a reverse scale, i.e., from 5 to 0, for food groups presumed away from the Mediterranean Diet; a composite score was then calculated, with greater scores suggesting higher adherence to the Mediterranean Diet [29].

#### Eating-related indicators

An *eating protective profile* was determined based on participants’ answers on three specific indicators of eating-related habits (i.e., eating breakfast regularly, not skipping meals, and low consumption of fast food). A composite score was calculated by averaging the scores on these three items, answered in a 4-point Likert scale ranging from never to six or more times/week [30].

Cognitive eating restraint was assessed with three items “I usually make an effort to eat less than I want”; “When I have eaten too much, I eat less than usual the following days” (indicative of a flexible approach to eating restraint) and “The only way I can control what I eat is to follow a fixed plan and not depart from it” (reflecting a rigid approach of eating restraint), from the Three-Factor Eating Questionnaire R-21 and the Rigidity of Attitudes Regarding Personal Habits Scale [31, 32]. A total eating restraint score was calculated by averaging these three items.

Reliance on internal hunger and satiety cues, an indicator of an individual’s trust in his/her physiological signals, was measured using two items (“I trust my body to tell me when to eat” and “I trust my body to tell me how much to eat”) from the Intuitive Eating Scale-2 [33].

Eating for non-emotional reasons, i.e., using food to satisfy physical hunger instead of to cope with emotional distress, was measured with a reverse-coded item (“I find myself eating when I’m feeling emotional (e.g., anxious, depressed, sad), even when I’m not physically hungry”), from the Intuitive Eating Scale-2 [33].

A total intuitive eating score was calculated by averaging the scores on reliance on hunger and satiety cues items, eating for physical reasons item, and cognitive eating restraint ( “I usually make an effort to eat less than I want”; reversed).

Eating induced by external cues was assessed via two items: “Being with someone who is eating, often makes me want to also eat” and “When I smell appetizing food or see a delicious dish, I find it very difficult not to eat – even if I’ve just finished a meal”, from the Three-Factor Eating Questionnaire R-21 [31].

The power that food has over the individual was measured with two items: “I think I enjoy eating a lot more than most other people” and “It seems like I have food on my mind a lot”, from the Power of Food Scale [34].

Uncontrolled eating was assessed with one item: “Sometimes when I start eating, I just can’t seem to stop”, from the Three-Factor Eating Questionnaire R-21 [31].

Participants answered all these items in a 5-point Likert scale ranging from Strongly Disagree (1) to Strongly Agree (5). Greater values signal greater levels on that eating indicator.

### Statistical analyses

Data analyses were performed using the IBM SPSS software (version 28.0), including descriptive statistics, bivariate correlations, and partial correlations with sedentary domains as the independent variables and the diet/eating related outcomes as the dependent variables, controlling for age, sex, BMI, and PA level. For the statistically significant correlations, linear regression models were performed using R, which are displayed in the Figures. Comparisons between sex, age tertiles, and BMI categories (normal vs excess body weight) were performed by One-way ANOVA for all the included variables. A 5% significance level was adopted.

## Results

The final sample included 301 adults (60.1% women), with 64.8% students, 21.9% teachers, and 13.3% staff. Age ranged from 18 to 72 years, with a mean age of 34.5 years. Participants spent an average of 404.5 (188.6) min/day in SB at work/school, 212.9 (146.0) min/day in leisure-time SB, and 71.9 (58.5) min/day in transport-related SB.

As presented in Table 1, participants with a BMI ≥ 25 kg/m^2^ showed a higher consumption of red meat (and products) and poorer eating indicators (e.g., higher external eating, uncontrolled eating, and power of food, and lower intuitive eating). Participants in the 3rd age tertile tend to display lower levels of PA but concurrently lower SB in all domains and more positive eating patterns (i.e., a protective eating profile with more breakfast consumption, less meal skipping and fast-food consumption; less uncontrolled, external eating and power of food; and a higher adherence to the Mediterranean diet with higher consumption of fruits, vegetables, legumes, fish (and fisheries), olive oil and less red meat). Finally, men tend to have more intuitive eating and eating for non-emotional reasons and less external eating than women, and in terms of Mediterranean diet adherence, some mixed results were found (i.e., higher consumption of non-refined cereals and legumes, but in opposition a higher consumption of red meat and alcoholic beverages, and a lower use of olive oil in cooking). No differences between men and women were found for any SB domain, but men presented significantly more PA than women.

**Table 1:** Comparisons between BMI, age, and sex categories, for the main variables (mean ± SD)

**Variables**	BMI < 25.0 (*n* = 199)	BMI ≥ 25.0 (*n* = 102)	Age 1st tertile (18–22 years)	Age 2nd tertile (23–41 years)	Age 3rd tertile (42–72 years)	Women (*n* = 181)	Men (*n* = 120)
**Demographics**							
Age (years)	32.83 ± 14.02	37.84 ± 13.2 **	19.80 ± 1.4	31.01 ± 5.8	51.04 ± 6.8 ***	32.93 ± 13.3	36.78 ± 14.6 *
Height (m)	1.68 ± 0.08	1.70 ± 0.11 *	1.68 ± 0.10	1.69 ± 0.09	1.69 ± 0.09	1.64 ± 0.07	1.76 ± 0.06 ***
Body mass (kg)	61.7 ± 9.2	84.8 ± 16.7 ***	65.2 ± 14.8	69.1 ± 16.6	73.6 ± 16.5 **	63.2 ± 13.6	78.9 ± 15.7 ***
BMI (kg/m^2^)	21.8 ± 2.2	29.1 ± 4.9 ***	23.0 ± 4.2	24.0 ± 5.2	25.6 ± 4.6 ***	23.5 ± 4.7	25.4 ± 4.8 ***
**Eating-related indicators (min–max)**							
***Mediterranean Diet Adherence***							
Non-refined cereals (1–6)	2.51 ± 1.1	2.51 ± 1.1	2.51 ± 1.1	2.59 ± 1.1	2.43 ± 1.1	2.40 ± 1.0	2.67 ± 1.2 *
Potatoes (1–6)	2.89 ± 1.0	3.00 ± 1.2	3.02 ± 1.0	2.90 ± 1.0	2.88 ± 1.1	2.91 ± 1.1	2.97 ± 1.0
Fruits (1–6)	3.55 ± 1.3	3.52 ± 1.3	3.25 ± 1.2	3.47 ± 1.2	3.87 ± 1.3 **	3.50 ± 1.2	3.60 ± 1.3
Vegetables (raw or cooked) (1–6)	3.23 ± 1.2	3.30 ± 1.2	2.86 ± 1.1	3.30 ± 1.2	3.57 ± 1.2 ***	3.22 ± 1.3	3.32 ± 1.1
Legumes (1–6)	3.21 ± 1.2	3.35 ± 1.3	3.00 ± 1.2	3.43 ± 1.3	3.30 ± 1.1 *	3.09 ± 1.2	3.50 ± 1.2 *
Fish (and fisheries) (1–6)	3.28 ± 1.2	3.33 ± 1.1	3.13 ± 1.2	3.13 ± 1.2	3.63 ± 1.1 **	3.31 ± 1.2	3.28 ± 1.1
Red meat and products (1–6)	2.20 ± 1.2	2.54 ± 1.3 *	2.63 ± 1.4	2.21 ± 1.3	2.14 ± 1.3 *	2.06 ± 1.2	2.72 ± 1.3 ***
Poultry (1–6)	2.46 ± 1.3	2.58 ± 1.3	2.69 ± 1.4	2.49 ± 1.3	2.37 ± 1.2	2.41 ± 1.3	2.65 ± 1.4
Use of olive oil in cooking (1–6)	5.10 ± 1.2	4.99 ± 1.3	4.69 ± 1.4	5.08 ± 1.3	5.37 ± 0.9 ***	5.22 ± 1.2	4.81 ± 1.3 **
Alcoholic beverages (1–7)	1.51 ± 0.9	1.63 ± 1.0	1.57 ± 1.1	1.47 ± 0.9	1.61 ± 0.8	1.40 ± 0.7	1.78 ± 1.2 ***
***Eating-related profile***							
Breakfast consumption (1–4)	3.52 ± 0.9	3.51 ± 0.8	3.40 ± 0.9	3.44 ± 0.9	3.71 ± 0.7 *	3.56 ± 0.8	3.46 ± 0.9
Meal skipping (1–4)	1.55 ± 0.8	1.57 ± 0.8	1.62 ± 0.9	1.66 ± 0.8	1.38 ± 0.6 *	1.56 ± 0.8	1.54 ± 0.8
Fast-food consumption (1–3)	1.59 ± 0.6	1.69 ± 0.6	1.86 ± 0.5	1.56 ± 0.6	1.48 ± 0.5 ***	1.63 ± 0.6	1.62 ± 0.6
**Eating behavior indicators (min–max)**							
Reliance hunger/satiety cues (1–5)	3.21 ± 1.13	2.61 ± 1.1 ***	3.01 ± 1.2	3.14 ± 1.1	2.87 ± 1.2	3.00 ± 1.2	3.01 ± 1.1
Eating non-emotional reasons (1–5)	3.38 ± 1.5	2.66 ± 1.6 ***	3.00 ± 1.6	2.94 ± 1.5	3.45 ± 1.6 *	2.79 ± 1.5	3.66 ± 1.5 ***
Intuitive Eating (1–5)	3.38 ± 0.9	2.72 ± 0.9 ***	3.15 ± 0.9	3.12 ± 0.9	3.20 ± 1.0	3.02 ± 1.0	3.37 ± 0.9 **
External eating (1–5)	2.08 ± 1.0	2.43 ± 1.1 **	2.48 ± 1.1	2.13 ± 1.0	2.02 ± 1.0 **	2.31 ± 1.1	2.04 ± 1.0 *
Power of food (1–5)	1.88 ± 1.0	2.44 ± 1.2 ***	2.34 ± 1.2	1.97 ± 1.1	1.91 ± 1.1 *	2.11 ± 1.2	2.00 ± 1.1
Cognitive eating restraint (1–5)	2.28 ± 1.0	2.76 ± 0.9 ***	2.39 ± 1.1	2.39 ± 1.0	2.53 ± 1.0	2.45 ± 1.0	2.43 ± 1.0
Uncontrolled eating (1–5)	1.98 ± 1.2	2.45 ± 1.3 **	2.44 ± 1.4	2.11 ± 1.3	1.90 ± 1.2 *	2.24 ± 1.4	1.98 ± 1.1
**Sedentary behavior domains & PA**							
Leisure-time (min/day)	213.9 ± 150.7	210.2 ± 137.5	290.5 ± 167.1	200.9 ± 141.8	155.3 ± 89.8 ***	212.1 ± 151.3	214.1 ± 138.1
Transport (min/day)	73.8 ± 57.5	68.5 ± 61.0	89.2 ± 64.9	67.8 ± 63.5	60.6 ± 42.1 **	67.9 ± 56.0	77.9 ± 61.9
Work (min/day)	412.5 ± 194.1	387.8 ± 177.7	439.3 ± 197.7	421.1 ± 218.6	356.8 ± 131.4 **	414.0 ± 196.8	390.1 ± 175.3
Total PA (min/week)	696.5 ± 620.4	604.6 ± 539.0	807.4 ± 717.8	671.0 ± 588.3	541.6 ± 441.1 **	612.8 ± 553.5	752.3 ± 648.3 *

The means and SD for the eating-related indicators and behavior traits are in relation to the corresponding response Likert scales

*Abbreviations: BMI* Body mass index, *PA* Physical activity

*p < .05, ** *p* < .01, *** *p* < .001 for the One-way ANOVA comparisons between categories of BMI, age, and sex

Correlations of SB domains and PA level with eating-related indicators for the overall sample are depicted in Table 2. Regarding eating habits, higher transport-related SB was associated with lower intake of fruits (*r* = -0.17; *p* = 0.003) and vegetables (*r* = -0.18; *p* = 0.001), lower use of olive oil (*r* = -0.16; *p* = 0.005), lower breakfast consumption (*r* = -0.20; *p* < 0.001), and higher intake of potatoes (*r* = 0.12; *p* = 0.042), higher fast-food consumption (*r* = 0.12; *p* = 0.042), which was translated into a lower adherence to the Mediterranean diet (*r* = -0.20; *p* < 0.001) and a less protective eating profile (*r* = -0.22; *p* < 0.001). A similar pattern of associations was observed for leisure-time SB: negative associations were observed with fruits (*r* = -0.16; *p* = 0.005), vegetables (*r* = -0.12; *p* = 0.037), and fish consumption (*r* = -0.16; *p* = 0.006), use of olive oil in cooking (*r* = -0.20; *p* < 0.001), and breakfast consumption (*r* = -0.21; *p* < 0.001); positive associations were found with fast-food consumption (*r* = 0.27; *p* < 0.001), red meat and products (*r* = 0.12; *p* = 0.040), and meal skipping (*r* = 0.16; *p* = 0.005). These results were also translated into a lower adherence to the Mediterranean diet (*r* = -0.20; *p* < 0.001) and a less protective eating profile (*r* = -0.31; *p* < 0.001). No significant associations were observed for the work-related domain. After adjusting for sex, BMI, and PA level (only for SB domains), similar results were obtained.

**Table 2:** Correlations of SB domains and Total PA with eating-related indicators and eating behavior traits

Variable	SB transport	SB work	SB leisure-time	PA level
**Eating-related Indicators**				
***Mediterranean Diet Adherence***				
Non-refined cereals	-0.10	-0.01	0.02	0.00
Potatoes	0.12*	0.03	0.10	0.06
Fruits	-0.17**	-0.02	-0.16**	0.16**
Vegetables	-0.18**	-0.03	-0.12*	0.07
Legumes	-0.07	0.01	-0.01	0.08
Fish (and fisheries)	-0.04	-0.01	-0.16**	-0.06
Red meat and products	0.10	0.09	0.12*	-0.01
Poultry	0.02	0.04	0.04	0.13*
Use of olive oil in cooking	-0.16**	-0.07	-0.20***	-0.09
Alcoholic beverages	0.03	-0.04	0.07	0.14*
*MedDietScore*	-0.20***	-0.05	-0.20***	0.00
***Eating-related profile***				
Breakfast consumption	-0.20***	0.01	-0.21***	-0.06
Meal skipping	0.11	0.05	0.16**	0.05
Fast-food consumption	0.12*	-0.02	0.27***	-0.05
*Protective eating profile*	-0.22***	-0.02	-0.31***	-0.04
**Eating Behavior Indicators**				
Reliance on hunger & satiety cues	-0.05	-0.03	0.04	0.01
Eating non-emotional reasons	0.10	-0.03	-0.05	0.11
Intuitive eating	0.09	-0.04	0.05	0.07
External eating	-0.04	0.01	0.09	-0.02
Power of food	0.03	-0.02	0.07	-0.05
Cognitive eating restraint	-0.12*	0.02	-0.11	0.04
Uncontrolled eating	-0.06	0.02	0.04	-0.00

*Abbreviations: PA* Physical activity, *SB* Sedentary behavior

* *p* < .05, ** *p* < .01, *** *p* < .001

Regarding eating behavior indicators, transport-related SB was negatively associated with cognitive eating restraint (*r* = -0.12; *p* = 0.031). No other significant associations were found. After adjusting for sex, age, BMI, and PA level, the association between transport-related SB with eating for physical rather than emotional reasons became significant (*r* = 0.13; *p* = 0.022).

Finally, higher PA levels were positively associated with fruits (*r* = 0.16; *p* = 0.007), poultry (*r* = 0.13; *p* = 0.029), and alcoholic beverages consumption (*r* = 0.14; *p* = 0.016). After adjustments, associations with poultry were no longer significant.

**Fig. 1:**
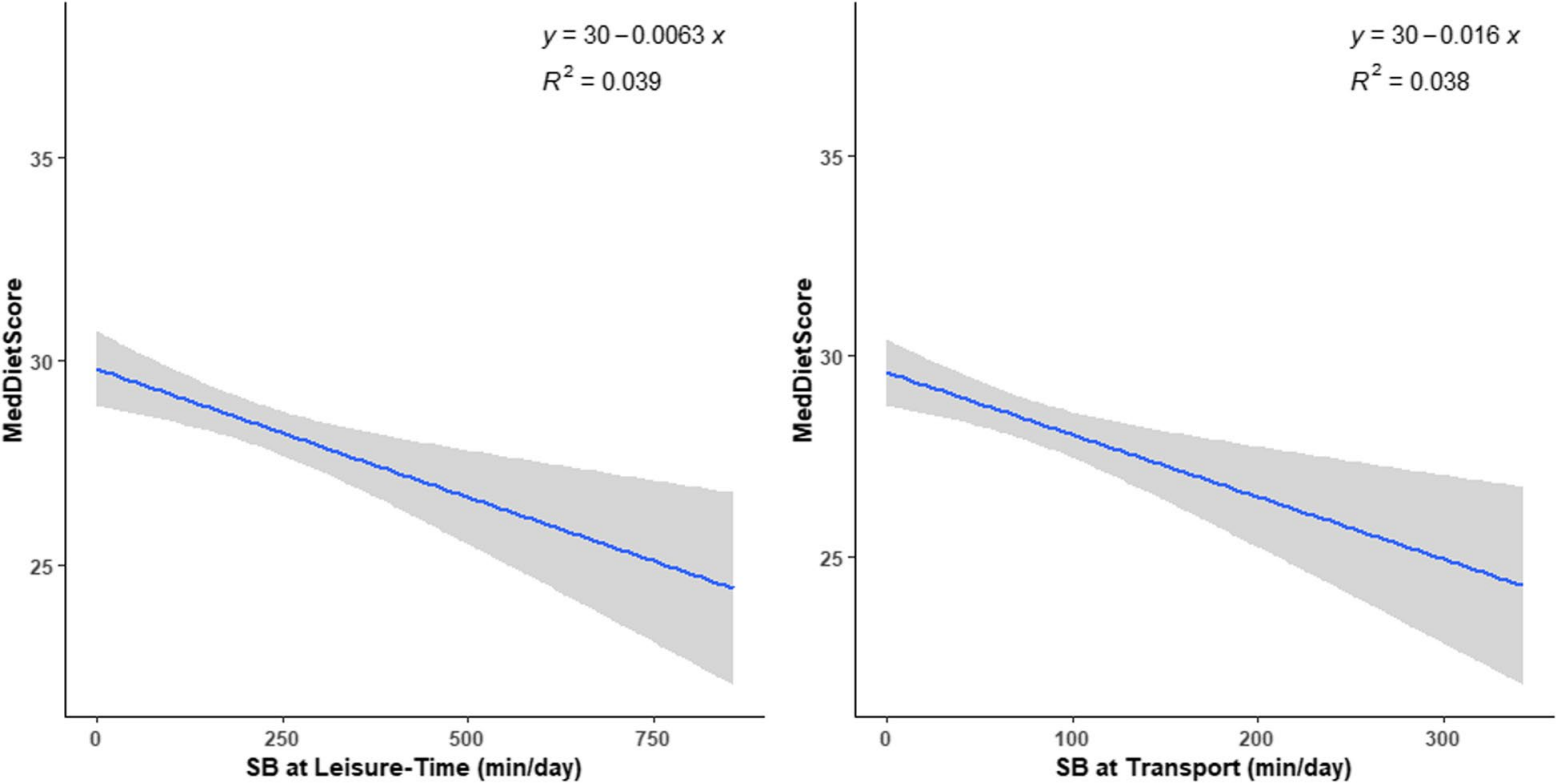
Daily time spent in transport-related SB and leisure-time SB vs. adherence to the Mediterranean diet

**Fig. 2:**
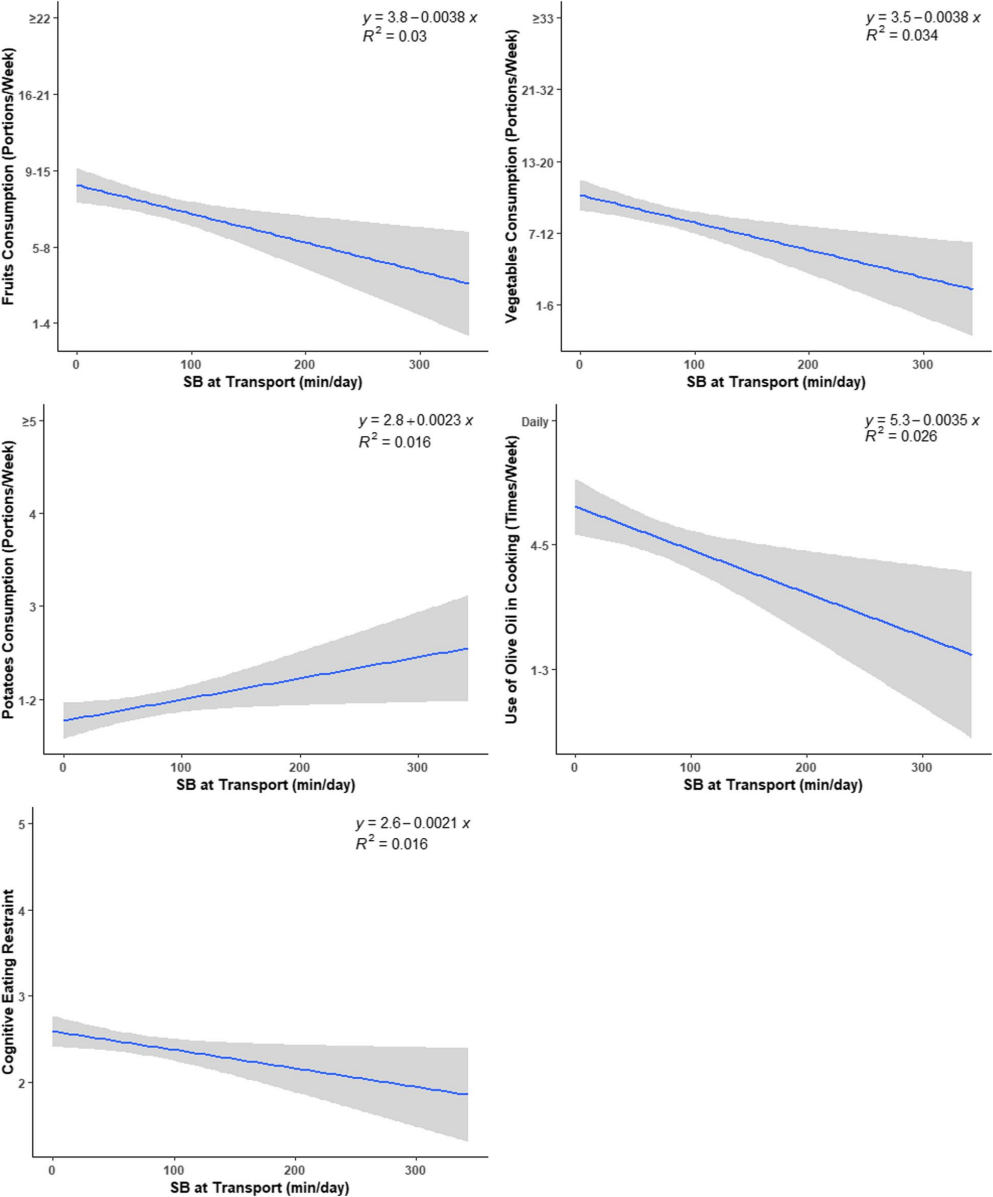
Daily time spent in transport-related SB vs. eating-related indicators and eating behaviors indicators

**Fig. 3:**
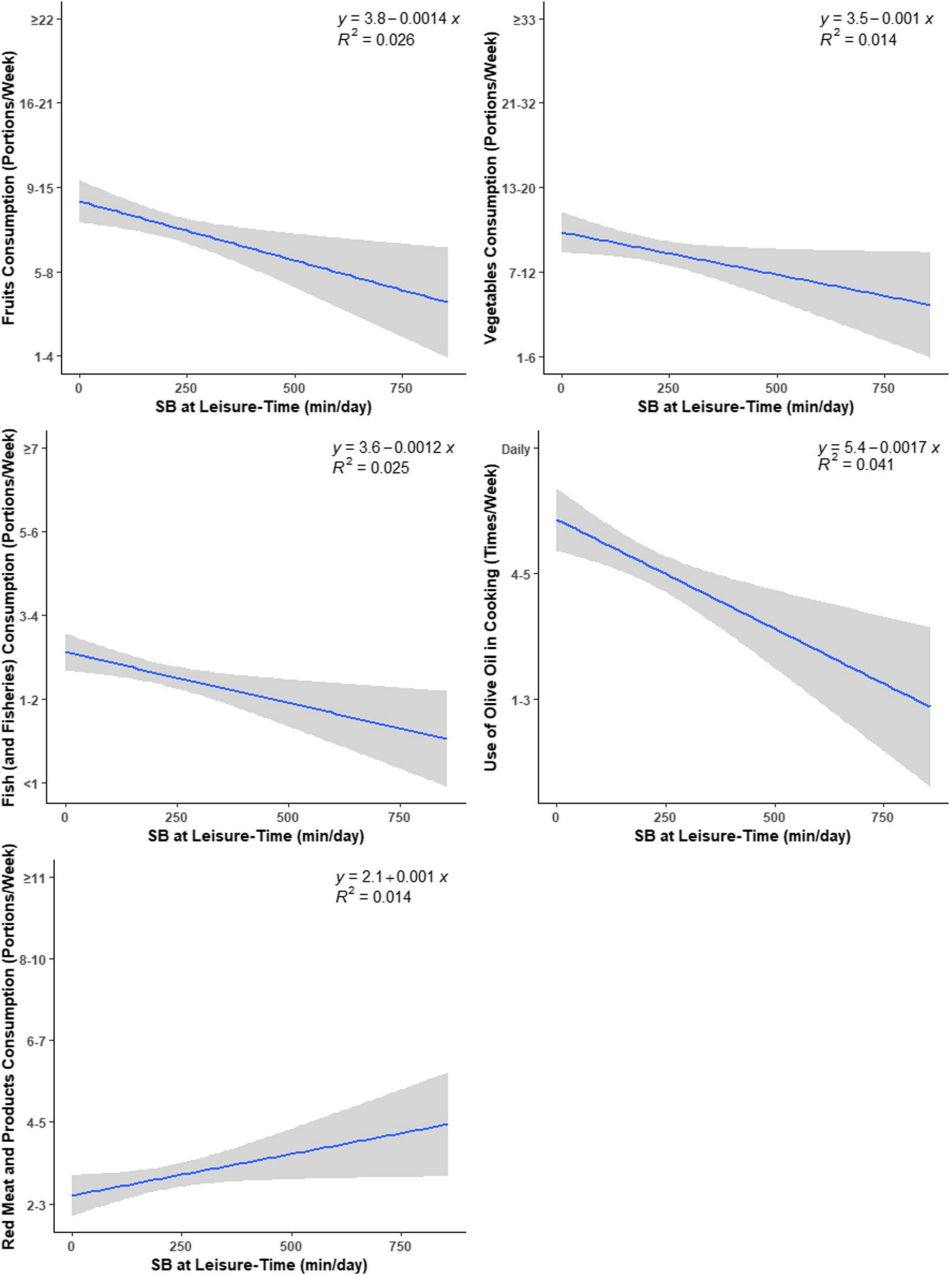
Daily time spent in leisure-time SB vs. eating-related indicators and eating behaviors indicators

The results for the significant associations in the overall sample are additionally presented in Figs. 1, 2 and 3.

Further exploring these associations stratified by age tertile revealed distinct associations across tertiles, as shown in Table 3. After the adjustment for sex, BMI, and PA level, these analyses revealed that in the first age tertile, higher transport-related SB was associated with higher intake of alcoholic drinks (*r* = 0.21; *p* = 0.048). In the second age tertile, a higher transport-related SB was associated with higher consumption of potatoes (*r* = 0.23; *p* = 0.022), and negative associations were observed with fruits (*r* = -0.21; *p* = 0.040), vegetables (*r* = -0.23; *p* = 0.021), and use of olive oil in cooking (*r* = -0.21; *p* = 0.034). These results were also translated into a lower adherence to the Mediterranean diet (*r* = -0.21; *p* = 0.040). For leisure-time SB, negative associations were observed with olive oil in cooking (*r* = -0.26; *p* = 0.009) and breakfast consumption (*r* = -0.33; *p* = 0.001); positive associations were found with fast-food consumption (*r* = 0.20; *p* = 0.046). These results were translated into a less protective eating profile (*r* = -0.25; *p* = 0.012). In the third age tertile, a higher transport-related SB was associated with a lower consumption of fruits (*r* = -0.21; *p* = 0.033) and less intake of alcoholic drinks (*r* = -0.20; *p* = 0.048); and higher leisure-time SB was associated with less fish consumption (*r* = -0.28; *p* = 0.004).

**Table 3:** Correlations of SB domains and Total PA with eating-related indicators and eating behavior traits, by age tertile

Variable	1st Age tertile				2nd Age tertile				3rd Age tertile			
	**SB transport**	**SB work**	**SB leisure-time**	**Total PA**	**SB transport**	**SB work**	**SB leisure-time**	**Total PA**	**SB transport**	**SB work**	**SB leisure-time**	**Total PA**
**Eating-related Indicators**												
***Mediterranean Diet Adherence***												
Non-refined cereals	-0.12	-0.11	0.03	-0.07	-0.14	-0.004	0.05	-0.13	-0.12	0.12	-0.04	0.23*
Potatoes	0.09	0.10	0.05	0.19	0.23*	-0.04	0.17	-0.10	0.10	0.08	0.16	0.08
Fruits	0.02	0.08	-0.09	0.26*	-0.21*	-0.06	-0.07	-0.00	-0.21*	0.09	-0.17	0.42**
Vegetables	0.02	0.08	0.06	0.09	-0.23*	-0.05	-0.07	0.00	-0.19	0.11	-0.08	0.27**
Legumes	0.14	0.07	0.14	0.00	-0.16	-0.03	-0.09	0.07	-0.18	0.05	-0.05	0.24*
Fish (and fisheries)	0.07	0.09	0.05	-0.06	-0.03	-0.03	-0.13	-0.03	-0.05	0.09	-0.28**	0.01
Red meat and products	0.02	0.12	0.11	0.03	0.10	0.03	0.06	-0.24*	0.07	0.17	-0.01	-0.00
Poultry	0.02	0.06	0.11	0.23*	-0.03	0.01	0.01	-0.05	0.04	0.06	-0.09	0.05
Use of olive oil in cooking	-0.01	0.02	-0.03	-0.06	-0.21*	-0.13	-0.26**	-0.08	-0.09	0.06	-0.03	0.10
Alcoholic beverages	0.21*	0.01	0.16	-0.02	-0.11	-0.13	-0.07	0.27**	-0.20*	0.19	0.08	0.18
*MedDietScore*	0.00	0.03	-0.05	0.01	-0.21*	-0.08	-0.17	-0.03	-0.20	0.09	-0.12	0.33**
***Eating-related profile***												
Breakfast consumption	-0.16	0.10	-0.01	0.14	-0.19	-0.02	-0.33***	-0.26**	-0.07	0.12	-0.04	0.09
Meal skipping	0.04	0.05	0.20	-0.04	0.09	0.03	-0.03	0.15	0.14	-0.12	0.14	0.05
Fast-food consumption	0.11	-0.09	0.14	-0.21*	-0.05	-0.14	0.20*	0.08	0.15	0.02	0.15	-0.19
*Protective eating profile*	-0.16	0.06	-0.19	0.19	-0.14	0.02	-0.25*	-0.26**	-0.18	0.12	-0.17	0.11
**Eating Behavior Indicators**												
Reliance on hunger & satiety cues	-0.05	-0.04	-0.03	-0.10	-0.08	-0.12	0.12	-0.05	-0.07	0.09	0.13	0.10
Eating non-emotional reasons	0.12	-0.13	-0.15	-0.06	0.17	0.03	0.05	0.09	-0.07	0.21*	0.14	0.27**
Intuitive eating	0.07	-0.21*	0.04	-0.09	0.14	0.03	0.07	-0.01	-0.03	0.12	0.21*	0.19
External eating	-0.04	-0.03	0.00	0.04	-0.15	-0.14	0.03	-0.09	-0.04	0.12	0.07	-0.01
Power of food	-0.04	-0.06	-0.08	0.01	-0.02	-0.06	0.05	-0.14	0.07	-0.06	0.07	0.00
Cognitive eating restraint	-0.17	0.20	-0.25*	0.09	-0.13	-0.12	-0.02	0.09	-0.04	-0.01	-0.07	0.04
Uncontrolled eating	-0.05	-0.01	-0.09	0.06	-0.15	-0.04	0.04	-0.06	-0.12	-0.01	-0.07	-0.01

Adjusted for Body Mass Index, Sex, and Total PA (only for SBs)

*Abbreviations: PA* Physical activity, *SB* Sedentary behavior

* *p* < .05. ** *p* < .01. *** *p* < .001

Regarding eating behavior indicators, after the adjustment for sex, BMI, and PA level, these analyses showed that in the first age tertile, a higher work-related SB was associated with less intuitive eating (*r* = -0.21; *p* = 0.043), and a higher leisure-time SB was negatively associated with cognitive eating restraint (*r* = -0.25; *p* = 0.017). In the third age tertile, a higher work-related SB became positively associated with eating for physical rather than emotional reasons (*r* = 0.21; *p* = 0.038), and higher leisure-time SB became positively associated with intuitive eating (*r* = 0.21; *p* = 0.036).

Regarding PA levels, after the adjustment for sex and BMI, these results revealed that in the first age tertile, higher PA remained positively associated with fruits (*r* = 0.26; *p* = 0.014) and poultry (*r* = 0.23; *p* = 0.025) consumption and became inversely related with fast food consumption (*r* = -0.21; *p* = 0.047). In the second age tertile, PA was positively associated with alcoholic drinks consumption (*r* = 0.27; *p* = 0.005) and became negatively related with red meat consumption (*r* = -0.24; *p* = 0.014), but also with lower breakfast consumption (*r* = -0.26; *p* = 0.008), thus a less protective eating profile (*r* = -0.26; *p* = 0.009). In the third age tertile, higher PA was positively associated with the consumption of non-refined cereals (*r* = 0.23; *p* = 0.018), fruits (*r* = 0.42; *p* < 0.001), vegetables (*r* = 0.27; *p* = 0.007), and legumes (*r* = 0.24; *p* = 0.015). These results were also translated into a higher adherence to the Mediterranean diet (*r* = 0.33; *p* = 0.001). A positive association between PA and eating for physical rather than emotional reasons (*r* = 0.27; *p* = 0.007) remained in the third age tertile after adjustment.

## Discussion

Research has explored the interplay between eating and movement behaviors, considering their potential interactional nature (i.e., changes in one domain may aggregate with changes in others) [35, 36], and that health-enhancing spill-over effects may arise when being more physically active and having a healthier eating pattern co-occur [6, 37], suggesting a pattern of protective versus deleterious associations [30]. However, contrarily to the wealth of evidence concerning the protective role of overall PA in this regard, limited research is available on the relationship between SB specific domains and eating-related indicators. Thus, this study sought to analyze the associations between distinct SB related domains (i.e., work, leisure-time, and transport) and several eating-related markers (i.e., food intake, protective eating habits profile, and eating behavior indicators) in adults.

Overall, findings extend the ones from previous studies focusing on PA and reporting a reduced preference for processed foods, red meat, fried foods, soft drinks, and snacking in more physically active individuals [38]. We found that SB, more specifically leisure-time SB, was consistently and negatively associated with fruits, vegetables, and fish consumption, use of olive oil in cooking, and breakfast consumption; and positively associated with fast-food consumption, red meat and products, and meal skipping, irrespective of sex, BMI category, and PA level. However, some of these results disappeared when adjusting for age, which means that these associations somehow vary with age. A more thorough exploration of these associations stratified by age tertile showed that these associations with the leisure SB domain were generally observed in the second age tertile (23–41 years-old), but less so in the youngest and oldest tertiles. We could not find any significant associations between work-related SB and eating-related indicators for the whole sample, but when stratified by age, we observed that this domain was negatively associated with intuitive eating in the youngest tertile (generally composed of students). Many students work in part-time and report high levels of stress, aspects that might explain this association. Poor time management has been reported as a barrier to a healthier eating style [39, 40] with a large proportion of university students perceiving their lifestyles as moderately-highly stressful and linked to the lack of proper time management [41]. Also, experiences of stress in university students (young adults) have been related to a poorer capability of eating in response to internal signals of hunger and satiety, which in turn, appears to result in higher emotional (less intuitive) eating [42]. This could also explain the negative association observed between the leisure SB domain and cognitive restraint in this age tertile. Future investigations are needed to replicate these analyses in varied samples (e.g., different cultures, with chronic conditions) and confirm these findings, given that this is the first study exploring associations between SB domains and eating-related indicators.

In line with previous evidence showing that lifestyle PA is positively associated with several eating behavior indicators [8, 9], our results showed that not only PA is favorably related with several eating behavior indicators amongst all age tertiles, as overall SB also seems to be associated with these outcomes, though in the opposite direction. These findings corroborate those found in a previous systematic review revealing significant associations between SB and elements of a less healthier diet including lower fruit and vegetable consumption, higher consumption of energy-dense snacks, drinks, fast food, and a higher total energy intake [16]. In addition, our study extends prior research by showing that not all SB domains appear to be associated with an unhealthier diet in the same way: leisure-time and transport related SB showed a greater number of associations of similar nature in the overall sample, while work-related SB did not show significant associations in the overall sample (only a significant association in the youngest tertile). Furthermore, after the adjustment for sex, BMI, and PA level, similar results were obtained, meaning that the associations were independent from these variables. The fact that transport-related SB was associated with a lower adherence to the Mediterranean diet in the overall sample may be potentially explained by one wasting more time commuting, and thus less time in leisure activities, which will possibly compromise the energy to prepare and cook meals at home. This is likely to contribute to a less healthy eating pattern by boosting take-away or home-delivered food (i.e., usually fast food). Indeed, transport-related SB was not only associated with a lower adherence to the Mediterranean diet, but also with a higher fast-food consumption.

Results by age tertile suggest that in the lower age tertile (mostly composed by students), transport-related SB was associated with higher alcoholic drink consumption. We believe that this association might be spurious, merely due to chance or related to an unforeseen confounding variable. Young adults tend to drink more socially and, at the same time, are more likely to spend more time commuting between home-university-social gatherings. It would be interesting to explore the influence of such factors in future analyses. Transport-related SB was also negatively associated with cognitive eating restraint, a potential risk factor for the adoption of dysfunctional eating patterns [43, 44], in the whole sample. Still, when stratifying analysis by age tertile, results were very diverse, not always coherent with the findings for the overall sample. We cannot ignore that data stratification ends up reducing the sample per group, which might explain the diverse patterns of association observed. Future studies confirming these findings in larger samples are therefore needed.

Regarding eating behavior indicators, transport-related SB was associated with lower cognitive eating restraint (i.e., tendency to restrict food intake to manage weight [45]), although these associations seem to depend on people’s BMI. One possible explanation for these findings might be related to the high percentage of our sample that commuted to and from work by using public transportation (approximately 45%). This type of transportation has been associated with greater steps and MVPA when compared to private transportation [46, 47]. It might just be that those using public transportation feel less demanded to control their eating, especially if they are not overweight, due to their potential higher PA level. On the other hand, we can speculate that transport-related SB may boost screen time during this period, resulting in a decreased cognitive impulse control and subsequent increase in brain activation for high energy dense foods, thus possibly contributing to a disinhibited vs. restricted eating pattern [22]. Screen time has also been associated with lower vegetables and fruits intake [19].

Work-related SB was not associated with any eating indicator in the overall sample, though associated with a less intuitive eating (i.e., less responsive to physiological hunger and satiety signals) in the youngest age tertile, composed of university students). These findings highlight that the deleterious role of SB may not only be related to SB itself (physiologically), but with participants’ age and lifestyle behavior profile. When considering other outcomes (e.g., mental health), this different pattern of associations by domain has also been observed. For example, leisure-time SB, characterized as mentally passive (e.g., watching TV), was found to increase the risk of depression in adults, while there was no harm associated with mentally active SB (e.g., using the computer at work/school) [48], therefore suggesting that not only the domain can play a role in the association between SB and mental health, as the specific type of SB within the domain may be of relevance. It may be the case that when leisure-time is not fulfilled with activities that nurture both body and mind, that may be a marker for risky health behaviors, such as a high sedentary time not occupied with any intentional activity triggering dysfunctional eating patterns and behaviors.

Indeed, excessive time in SB has been shown to be independently associated with several non-communicable diseases [13, 14], and even with higher mortality [13, 15]. Hence, there is a possibility that SB may play a specific role in people’s diet and eating behaviors, that can somehow alter the association between PA and these important outcomes (e.g., by limiting PA’s health-related outcomes). Future research would do well to explore these interactions.

Despite our focus on SB, we also explored the associations between PA and eating-related indicators and confirmed previous findings showing that higher PA levels were positively associated with healthier dietary choices [49], therefore reinforcing PA’s role as a gateway behavior for healthier eating. Unexpectedly, PA levels were not associated with any eating behavior indicator in the overall sample, but after adjustments, a positive association with eating for physical rather than emotional reasons emerged. This finding is in line with previous studies, reporting negative associations between PA and emotional eating [8, 9], further suggesting that age might be a relevant factor in this relationship. Post-hoc analysis, exploring these associations by age tertile showed that PA was associated with higher intuitive eating (i.e., in response to physiological hunger and satiety signals and not driven by emotional reasons) in participants from the oldest age tertile. In other words, age appears to be a protective factor against a more emotional (less healthy) eating pattern.

In today’s obesogenic food environment, regulating eating behavior is very demanding, with homeostatic drives involved in appetite control being easily superseded by hedonic, reward-based drives, that encourage eating beyond physiological necessity [50]. Also, emotional states have an important impact over one’s eating behavior, mainly by depleting his/her cognitive resources [51], which has in fact been associated with increased consumption of unhealthy, highly palatable foods [52]. In this context, the quality of food choices is determined by food hedonics, and deliberately and successfully resisting external and internal eating cues requires the identification of factors that can facilitate one’s eating self-regulation. SB, as PA, could be such a factor.

Important limitations of this study include its cross-sectional nature, which precludes us from inferring causality, and the nature of the sample – a convenience sample composed of students, teachers, and staff from the University willing to participate (perhaps showing a selection bias towards more motivated individuals). Another limitation concerns the self-reported nature of the variables under scrutiny, particularly diet, PA, and SB-related variables, which can be affected by recall difficulties and possible under-/overestimations. Although device-based measures of PA and SB would be preferred due to their accuracy, they may not capture the domain and context specific behaviors targeted in the current study. Thus, widely used, validated methods to measure PA and SB were selected, to balance the need for precision vs. feasibility. Finally, potential bias derived from a social desirability effect regarding body weight, eating-related indicators, PA, and SB levels, cannot be excluded.

The main analyses conducted in this study allowed us to explore the cross-sectional associations between distinct SB domains (i.e., work, leisure-time, and transport) and several eating-related markers (i.e., food intake, eating habits’ protective profile, and eating behavior indicators) in adults, while also exploring if these relationships were independent of sex, age, BMI, and PA levels. This was an underexplored research arena as very limited research addressing the associations between SB domains and eating is available, neglecting the potentially interactive effects of multiple diet components and related behaviors. Furthermore, evidence has been considering SB and eating traits as separate health risk factors, but our data brings novelty to this overlooked arena, by studying their inter-relationships, especially considering different domains of SB.

Taken together, our results highlight the importance of considering the complexity embedded in health behaviors. Sedentary behaviors should not only be considered in terms of their physiological impact. The potential health risk of this type of behavior lies on the behavioral health patterns involved and their psychological consequences. SB domains are, thus, of critical importance, also for eating behavior regulation, as demonstrated in other outcomes (e.g., mental health) [53]. Public health strategies should augment literacy on SB, namely on how SB can be accumulated via different settings. Furthermore, public health literacy efforts need to extend besides the more known deleterious effects of SB on health (e.g., diabetes, cardiovascular diseases) to also include the interplay with eating-related indicators.

Health literacy and public health campaigns devoted to promoting more active and healthier lifestyles may target SB domains of higher risk (i.e., leisure-time, and transport to some extent), and be tailored to different age groups. As a practical application from our findings, governmental bodies, health professionals, epidemiologists, teachers, parents, must be aware that not all SB may impact eating in the same way, and fortunately it seems that sedentary behaviors that we can somehow voluntarily modify are the most related with risky eating patterns. In sum, this information may assist public health authorities in focusing their efforts and strategies on specific domains, thus promoting active forms of commuting, and reducing SB in the leisure setting.

## Data Availability

The datasets used and/or analyzed during the current study are available from the corresponding author on reasonable request.
